# Address Delivered before the Graduating Class of the Baltimore College of Dental Surgery, March 1st, 1852

**Published:** 1852-04

**Authors:** Eleazar Parmly


### ARTICLE II.

*Address Delivered before the Graduating Class of the Baltimore College of Dental Surgery, March* 1*st*, 1852. By Eleazar Parmly.

Gentlemen Graduates:

I feel happy in congratulating you on the completion of your collegiate course, and in expressing to you the high satisfaction which I feel, in common with other members of the examining committee, at the very creditable manner in which you have earned your degree.

Neither the duty nor the pleasure of addressing you on this occasion, was originally assigned to me, nor does it belong to my particular office, but permission having been granted to me, I ask your particular indulgence, while I make a few remarks on the subject of dental education, as connected with dental and medical colleges. I am led to do this in vindication of my own course, and that of the institution from which you now graduate; and also as an act of justice to your professors—to the former graduates, and to the gentlemen of the examining committee. These latter gentlemen have been appointed, from year to year, to bear testimony to the professional practice and principles taught in this institution—to the various courses of instruction and to the finished specimens of artificial manufacture, as well as of the higher operations in dental surgery, of those who are deemed worthy to receive the diploma of the college.

The necessity which has made this duty at this time imperative, may be found in some remarks in an article in the New York Journal of Medicine, one of the most valuable and widely circulated periodicals devoted to medicine, and the collateral sciences published in this country.

The title of the article to which I allude, is "Dental Education," its author, John Trenor, M. D., dentist, of New York.

Dr. T., after very largely and very properly setting forth the necessary qualifications of dentists, to insure to them complete success in professional practice, and also in the evils which have existed, and still exist, in regard to the imperfect manner in which, by far the largest portion of dental practitioners have been educated, uses, in relation to the dental colleges, the following words:

"Under the plea of remedying all these evils, what are termed dental colleges have been recently brought into existence. Conscious of the wants in this branch of the medical profession, and of the obvious inefficiency of a large number of those who appear in the capacity of its practitioners, and a belief taken, if not altogether for granted, certainly without sufficient investigation, that these institutions must necessarily remedy the deficiencies so generally felt and justly complained of, some of the members of the medical profession have accorded to them a degree of countenance and approbation, to which it can be easily shown, that they are by no means entitled. They come before the public with such confident promises and plausible pretensions, and as at present constituted, are so decidedly inefficient, that they are a greater drawback to improvement than if they had never existed. They profess to remedy an evil, which they most effectually and glaringly magnify. They hold out the idea of giving a complete and finished course of instruction on dentistry, while full two-thirds of what should be taught, and that the most important too, viz. all the instruction which every medical school inculcates in medicine and surgery, it does not enter into their arrangements, nor do they possess the ability, with any degree of usefulness or benefit, to perform."

Now such language applied to this college is either true or false. If true, it should be sustained by stronger evidence than bare assertion; if false, let it be thrown back upon him from whom it emanated. It is a fact, that he who published this to the world, has never entered these walls, nor does he know any thing, by personal examination, of the system of instruction here pursued, and it is equally true, that if he had been acquainted with its various courses of instruction, as a man of common courtesy, of honor and honesty, he could not have uttered such sentiments. Dr. T. has unnecessarily treated with personal insult and indignity all who have been connected with, or interested in, this institution. As the Baltimore College of Dental Surgery is the only one of the kind that has until lately had a name or an existence in the world, and the only one that has been acknowledged, sustained and approved by medical men, we are particularly called upon to understand that this is the college at which this shaft has been directed, and its professors and teachers are the men who are charged with being instrumental in establishing the "greater drawback," and in retarding the progress of professional improvement. But more than all this, those distinguished medical gentlemen who have been chosen as a committee, from year to year, from among the most eminent practitioners of this city, and who have patiently attended upon the examinations, are "*the members of the medical profession*," to use the language of Dr. Trenor, "*who have accorded to them a degree, countenance and approbation to which it can easily be shown that they are* (the colleges) *by no means entitled.*"

Had Dr. T. visited this school, and here witnessed the weakness, imbecility, and error, which he would fain ascribe to it; and had he, from careful observation, attained to an honest belief of what he asserted, he would have had some excuse for his sweeping denunciation, though, even then, no warrant for the harsh expressions he has used. But as it is, evidently without the slightest knowledge of the studies pursued in this college, its discipline, its order, or its exercises, he has charged inefficiency upon the whole system, and questioned the judgment and candor of some of the most distinguished physicians of Baltimore, and dentists of America, who, by testifying to the qualifications of the graduates, have given their "*countenance and approbation*" to this school.

When I speak of the eminence of those medical gentlemen, I wish to be particularly understood as intending to declare that they stand upon the highest plane of their profession, side by side, with the most eminent men of other cities, and of other lands. And these are the men who have aided "*most effectually*" the efforts of this college to "*glaringly magnify*" the evils of ignorance, quackery and presumption, if I may be allowed to repeat, in this assembly, the disrespectful language that has been applied to the professional gentlemen of Baltimore. This, besides being uncourteous, is untrue, for it cannot be proven that any other than the best principles and practice, and only the best known to dental surgery, have been taught here.

Dr. T. may not be aware of it, but we assert it, and challenge successful contradiction, that all the evils that have resulted or ever will result from the practice of all the graduates of this, and other dental colleges, that teach the same principles, will never equal the *glaring* one which he daily *magnifies* in recommending and using mercury and silver as a better stopping for teeth than gold, and professors who teach this amalgam doctrine, and amalgam practice in medical colleges will do infinitely more harm than good to the students, to the profession and to the public. Certainly the medical colleges who appoint such as lecturers or professors to teach dental surgery in their institutions, will "*accord to them a degree of countenance and approbation to which it can be easily shown that they are by no means entitled.*"

As I may not have another opportunity, I will avail myself of this, to express on my own behalf and on behalf of the college, our grateful thanks for the "*countenance and approbation*" so often and so generously bestowed upon this institution by the gentlemen in question; and the strongest proof that I can give of my confidence in the system of its instruction, may be found in the fact, that notwithstanding we have medical schools in New York, which compare with the best institutions in the country, and notwithstanding I can furnish under my own roof as many facilities in the practice of dental surgery and mechanical labor, as can be found in any other private dental establishment, I have sent my only son and other members of my family to the Baltimore College of Dental Surgery, for the purpose of securing to them better and more complete instruction than any one individual can impart.

To this, I may add, that I have heard dentists and medical men, on our committees of examination, professors in medical colleges of highest rank, who stand as erect, morally and professionally, and who are, in every way, as capable of judging in the matter as those who have never been here, and know but little or nothing of our policy, declare, after "*sufficient investigation*," that the system of instruction hitherto pursued in this college, is the most perfect that has ever been offered to the dental student; and we have good reason to believe that other colleges, founded on the same basis, will fully sustain "*the confident promises and plausible pretensions*" set forth in their annual announcements.

It is, however, a gratifying evidence of the scientific progress of the present day, that dental instruction, embracing a general knowledge of the structure and diseases of the teeth, is engaging the attention of some of the most popular medical schools of our country.

As we have anxiously desired, for more than a quarter of a century, "a consummation so devoutly to be wished," I shall confine the few remarks I have now to make, particularly to its relations as far as they affect the interest of the profession, and it becomes peculiarly appropriate as I regard it as decidedly the most auspicious "sign of the times" in favor of that class of institutions of which the Baltimore College of Dental Surgery is the *pioneer*.

It is worthy of remark, that the fortunate event of which we now speak, has been the direct or indirect result of the efforts of one, who may differ widely in opinion from us, in relation to the necessity of dental colleges, or institutions of practical learning. Whether his only aim at first was to do entirely away with this class of schools, by substituting what he conceived to be a better system of dental education, at the same time raising the character of the profession to a level with other departments of medical and surgical science, I know not: but believing this to be the case, I respect both the motive and the mover of this propitious change.

The proposition when published, having been well received, it was soon followed up by another member of the profession, with a zeal and earnestness which, with sufficient power, would not only have swept from existence our edifices themselves, but would have visited with obloquy and reproach, all who have, in any way, extended to them their "countenance and approbation." This latter effort may possibly have a very different effect from what it was anticipated, and should it even prove to be so, it will not be the first example in the history of science, of the unintentional advancement of the interests of truth, by its secret or avowed enemies.

It is not necessary to waste either time or words in enumerating reasons which may be supposed to have operated with our friends or our enemies, to induce them to appear in argumentative opposition to a class of modern institutions, chartered by the legislatures of several states, for the special education of a numerous profession, essential to the welfare of civilized society. If their motives have been benevolent, as we have no disposition to call in question, they will not fail of the reward—an approving self-respect.

But whatever may have been their promptings, the result has been auspicious to the permanent well-being of the dental profession throughout our land ; and will establish the necessity for the continuance of the schools that now exist, or will call into existence, in some other way, a system of instruction, embracing all that is now taught in them, for I affirm, and fear not contradiction, that practical dentistry cannot be acquired so perfectly in any other way, as in a system similar to that practiced in this school and adopted by others.

There are in the United States, twenty-seven medical colleges, not quite one for each state. Twenty-seven dental lecturers, or one in each of these schools, would produce the most salutary effect in elevating the character of the dental, and impressing the medical, profession with the importance of the subject.

The arguments that have been employed by some of our professional co-laborers in medical and dental journals of a recent date, have already led to the institution of at least one dental lectureship in one of the medical schools of New York. During the past autumn, Dr. C. C. Allen was employed to lecture to a class of medical students in the New York Medical College, and more recently it has been understood that there will be established a dental professorship in the College of Physicians and Surgeons of the State of New York.

These instances alone, as good beginnings, we hail with pleasure, and the sooner such precedents are followed by other medical institutions, the better it will be for the profession, for the public at large, and for those special colleges established for the education of practical dentists. We say *practical* dentists, because we regard it as one of the wildest chimeras of the age, to suppose for a moment that a practical education in the difficult art of dentistry can ever be attained in the class lecture rooms of the best medical college in the world.

It has been asserted in a quarter, from which truth on this subject ought to emanate, that the dental practitioner cannot be fully qualified for the duties of his calling, without a thorough knowledge of medicine and surgery, such as dental colleges are not qualified to impart. The fallacy of this position is plainly manifest without an argument, and scarcely needs the antidote of a sober denial. It possesses not a whit more truth than the parallel proposition, that the medical and surgical practitioner cannot exercise his calling successfully, without a more perfect knowledge of the dental science than the medical colleges are prepared to impart. If the former position destroys the necessity of dental colleges, the latter equally denies the utility of all schools of medicine and surgery.

Yet will the universal appointment of dental lecturers in medical schools be welcomed with honorable pride by every high minded member of our profession ; for what can more directly elevate the theory of dental science to its merited position, among the kindred sciences, than to find its collegiate professorships working side by side with those of general anatomy, medicine and surgery ?

Well may we say, that in our favorite calling, the present is an age of progress ; for in truth, the most ambitious members of the dental profession neither ask nor desire any higher exaltation, than to stand upon an equality with the oculists, the aurists, the surgeons, and the physicians, who receive their elementary and preparatory instruction in the medical schools of the age ; and this will be their position when all the schools of general surgery and medicine shall find it expedient to annex to their professorships the chair of dentistry.

Nor can such a policy fail to become necessary to the prosperity of even the secondary grade of medical colleges, as soon as those of the first class in the larger cities shall have fairly set the example. Let it then be hailed as a matter of the most sincere congratulation among the members of our profession, as well as among the dental colleges now established in several of the United States, that the medical colleges of New York and Philadelphia are rapidly moving in the direction of this most desirable object. If New York seems to have taken the lead in this movement, it is probably more in appearance than reality, inasmuch as Philadelphia was first to agitate the question.

This she did, not only by the friends and conductors of the medical colleges, but most effectually by one of the justly celebrated dentists of that city, Dr. E. B. Gardette. This gentleman, of whose professional skill, educational accomplishments, and personal excellences of character, there is little danger of speaking in terms of unmerited praise, has taken unwearied pains to convince the faculties of the several American colleges of medicine and surgery, of the great utility of dental professorships in those institutions of learning.

So far as Dr. Gardette's well-considered arguments demonstrate the importance of such professorships, we admit, most frankly, the full force of his reasoning ; but we have been unable to discover any proof that such professorships preclude, in any manner, the separate necessity of colleges, or of some other adequate means of practical instruction. For "*it can be easily shown*," that medical colleges at best, can only best qualify students for entering upon a practical course in "*what are termed Dental Colleges.*"

The reasons of this difference in the results of his argumentation, will manifestly, we trust, appear in the sequel.

It must be clearly evident to all sensible men, that the advantages to be derived from the lectures of a professor of dental science in an ordinary medical college, are entirely distinct and different from those of a class of professors and teachers in a dental college, where practice is blended with theory in every step of the student's progress. Particularly will this be manifest to persons familiarly acquainted with the methods of instruction in these two classes of institutions, of which number should be all those who undertake to disparage dental colleges, when compared with those of general surgery and medicine.

Nothing can be more certain than that medical schools will introduce nothing in the way of instruction which does not meet the wants of all classes of their students, as well those who are to be physicians and surgeons, as those who are to be dental operators in after life. Hence it follows, that the trustees of those medical colleges which may have instituted lectureships in dental science, are fully convinced that all these classes of students need a certain amount of reliable information in relation to the structure and diseases of the teeth. They all need this knowledge, because it pertains legitimately to all departments of the healing art; and if such general knowledge of dentistry were not common to the necessities of all these classes of students, it would be evidently absurd to waste upon it the time and treasure of medical students.

In dentistry it is the province of the brain to direct, the hand to execute, both must work together, and as much care is required in educating the one as the other; and he who has any adequate knowledge of dental practice, or of dentistry as an *artistic science*, in which the *science* alone is of no practical utility without the *art*, needs not to be informed that a dental student coming to the duties of his profession with no other preparation than such as he could gain in common with other pupils of medical schools, would be about as fit for his business as would be the commander of a ship who studied navigation in a country academy, and who had never seen even a ship or the ocean, or a merchant who had attended a few lectures on penmanship, commerce, arithmetic and free trade.

There can be little doubt that a promiscuous assemblage of young gentlemen, destined for the various avocations of human society, and engaged in their educational studies, might derive some benefit from a well composed lecture from a master of his subject on either navigation, penmanship, arithmetic, or commerce; but the question still recurs, is this *the best*, or even a practicable, mode of teaching thoroughly these branches of learning? Has it ever been known in any age or nation, that a man has been taught to write a handsome business hand with his right hand in his pocket, listening to the mere verbal instructions of his teacher? And yet we hazard little or nothing in the assertion that the difficulty of writing a good business hand is not for a moment to be compared with that of becoming an expert, well instructed and elegant operator in dental surgery, or even in what has been inappropriately called dental mechanics. It is not, we think, too much to say, that of every thousand individuals it would be more probable that fifty would become good penmen under competent masters, than that *one* only should become an accomplished dental operator under the best possible instruction. To those most familiar with the subject, who have witnessed for years the wretched operations of amalgam dentists, first "medically educated," this will be seen not to be taking too high a ground for the profession which we exercise.

That amalgamists should recommend medical colleges as the best sources of gaining professional knowledge, causes no surprise, for there they can, in a single lecture, and a short one too, from the professor of chemistry, learn not only the compounding, but the nature, quality, and effects of amalgam, besides its "*artistical employment*"—but skilful dentists who have obtained their knowledge and skill by hard labor, know that it is just as impossible to become a good operator on teeth, from hearing lectures, without using the hands in dental manipulations, as it would be for a man to become a distinguished artist by reading the lectures of Sir Joshua Reynolds, or the life and early history of Sir Benjamin West.

But, perhaps, it may be said that a system of as thorough instruction in practice as well as theory, may be instituted in the ordinary colleges of medicine and surgery, as those now devoted exclusively to dental education—but this is yielding the argument, and fully admitting all that we wish to prove. For then it instantly transforms a medical college into a college of dentistry, and we are not disposed to deny that after such a change one would be just as efficient as the other. But this is by no means the point at issue between us and the opposers of dental seminaries. They would fain persuade us to believe, that a simple lectureship in addition to those existing in medical schools, and such as has been recently established in New York, is better adapted to the purposes of dental education than such dental colleges as are now in operation in several of the American states. This is the point in question, and to this we hold our antagonist, or one of them at least, as being the very position which he has voluntarily assumed.

I value, appreciate and would most earnestly recommend to the dental practitioner the very highest degrees of scientific and professional attainment—but not to the exclusion of common civility and common respect towards those who do not come up to an assumed or a real standard of professional qualification.

There are five teachers in this school who embrace in their teaching, anatomy, physiology, pathology, therapeutics, including materia medica—the general principles of surgery, surgical operations of the head and neck, and the theory and practice of dental surgery proper.

If these several branches are not in this school and should not be in others as perfectly taught as they might be, the fault lies not in the system, but in the teachers, or may be with the students, and yet with the most perfect teaching and best students it is declared to be a one-third system. We will, therefore, for a moment, examine the justice of this remark, and bring in as evidence the labors of those who have given the highest dignity and the highest character to our profession.

We are familiarly acquainted with the finished qualifications for usefulness of a great number, all of whom commenced practice and rose to eminence with much less than one-third of the advantages furnished by this one-third system; and among such may be found the names of Hudson, Nasmyth, Waite, Cartwright, Koecker and Hayden.

I am constrained to make these remarks from the indignities so often shown to those whose good fortune it was not in early life to receive from charity, or otherwise a medical degree, from a medical college. And I would further say, that the complimentary or honorary degree conferred near the close of his life, of which so much has been said, upon the celebrated Hudson, relative, patron and preceptor of him who now stigmatizes dental colleges, was not conferred for his early studies in medicine and surgery, but for the eminent services and aid he rendered in elevating, by his labors, a branch of the healing art—and the bestowal, or the receiving of his diploma, added nothing to his professional merit, or to his professional qualifications.

Of all the distinguished men who have preceded us in our professional art, whose operations secured to them the greatest name, and to their patients the greatest good ; there was no one among them, as far as I am acquainted with the history of dentists, who was at the outset of his professional career "medically educated," but they were all those, I believe, who obtained their knowledge by personal study, observation, and practical experience. And we affirm, from knowledge and experience, that ten years so spent was not equal, in practical advantage, to two years in a school of dental surgery. The difference between the two systems is best seen and determined by comparing the rough and unfinished operations of the first class, whether medically educated or not, with the beautiful and finished productions that we have here witnessed from the hands of the graduates of a dental college.

We shall, therefore, conclude our remarks by condensing our opinions on this subject with the following simple propositions, to which we invite the attention and the criticism of all respectable gentlemen who differ with us in opinion on these matters.

First.—Students of general surgery and medicine do not need, and therefore will not seek any other than the most general principles of dental science, and, consequently, they would not assist in sustaining a complete course of practical and theoretical dental instruction in any one of those colleges.

Second.—If only one lecturer be sustained in each medical school, and if he confine himself to subjects equally important to all classes of his pupils, still the student in dentistry will be left to seek his practical education where best he can find it, and after all, to make his selection between private instruction and public dental colleges.

Third.—The advantages accruing to the dental practitioner from a general acquaintance with the principles of medicine and surgery are not only admitted, but positively declared and insisted upon by all the dental colleges, and their advocates, inasmuch as competent professorships in all kindred branches of the healing art, are actually established and sustained with zeal and talent in all these seminaries—by men, too, who have held corresponding professorships in medical colleges.

Fourth.—The past history of dental practice has demonstrated the fact, that the greater part of the truly successful members of the profession, almost without an exception, have derived the elements of their success from sources widely different from the sciences taught in the schools of medicine and surgery, however useful, we contend, those sciences must be admitted to be, in connection with their other qualifications.

Fifth.—There is not the remotest probability that a course of really practical instruction in dentistry, either surgical or mechanical, will ever be incorporated with the ordinary lectureships of medical colleges, and, in consequence, in spite of all argumentation on the subject, the student in dentistry will, at the close of his collegiate medical courses, when only he will be best qualified to enter a dental college, be left where he now is, to make his own selection between dental colleges and private instruction, or what is still more probable and desirable, that he will avail himself of the peculiar advantages of both, as do the students of law and medicine when they commence and complete their education in the offices of their private teachers.

Sixth.—The necessity of private tuition is at least partially obviated by the privileges which the students of this college enjoy in the infirmary connected with it. In the provisions of this valuable institution, the pupils of the Baltimore College of Dental Surgery are permitted to bear their part, and as in all other charitable or benevolent objects, to derive benefit and advantage to themselves whilst they do good to others.

During the session of this school, which closed in 1851, the number of distinct dental operations performed in the department of that institution appropriated to the students, amounted to upwards of seventeen hundred, and during the session which now terminates, to about an equal number.

This is a fact of very great importance in estimating the advantages enjoyed by students educated at this school, or at others having the same facilities.

If facts, like these which I have given, do not thoroughly substantiate the claims of dental colleges to the reputation of "*usefulness*" and "*benefit*" in professional education, then I have only to add, that *error* is as beautiful as *truth*, and slander more just than justly "*accorded countenance and approbation.*"

There is a great deficiency somewhere. If the faculty of this college, consisting of five professors, possess combined talent and knowledge equal only to one-third of what is assumed by Dr. Trenor, and if their combined efforts enable them to teach one-third only of what he individually professes to know and to be able to teach, and *that* one-third is only one-third of what it is "*most important*" for students to acquire, there is certainly a great want of talent, truth and ability, on the part of the professors. But if they do faithfully perform and fulfil all their "confident promises and plausible pretensions," and teach at least, *one-half* of what the Doctor is able to teach, and one-half of what it is "most important" for the student, in his preliminary course to learn, then, there is a great want of respect, truth and justice in Dr. T's remarks, and to you who have had ample opportunity of judging, I submit whether your professors, by an honest discharge of their obligations and responsibilities, and a faithful fulfilment of their "promises and pretensions," have rewarded you for the time you have spent, and for the pecuniary and personal sacrifices you have made in coming to Baltimore. Or whether they, by a total neglect of their duties, and by falsely assuming to teach what they do not "*possess the ability with any degree of usefulness or benefit to perform*," have deceived your hopes, disappointed your expectations, and despoiled you of your time and money. With you, my young friends, with every good wish for your health, prosperity and happiness, I leave the decision of the question.

